# Antibiotic Resistance of Bacteria Involved in Urinary Infections in Brazil: A Cross-Sectional and Retrospective Study

**DOI:** 10.3390/ijerph13090918

**Published:** 2016-09-15

**Authors:** Wellington Francisco Rodrigues, Camila Botelho Miguel, Ana Paula Oliveira Nogueira, Carlos Ueira-Vieira, Tony De Paiva Paulino, Siomar De Castro Soares, Elisabete Aparecida Mantovani Rodrigues De Resende, Javier Emilio Lazo-Chica, Marcelo Costa Araújo, Carlo José Oliveira

**Affiliations:** 1 Postgraduate Course in Health Sciences, Federal University of Triângulo Mineiro, 38061-500 Uberaba, MG, Brazil; wellington.frodrigues@hotmail.com (W.F.R.); camilabmiguel@hotmail.com (C.B.M.); jlazo@dcb.uftm.edu.br (J.E.L.-C.); 2 Institute of Genetics and Biochemistry, Federal University of Uberlandia, 38400-902 Uberlandia, MG, Brazil; anap812004@yahoo.com.br (A.P.O.N.); ueira@ingeb.ufu.br (C.U.-V.); 3 Faculty Morgana Potrich—FAMP, 75830-000 Mineiros, GO, Brazil; wellingtonrodrigues@fampfaculdade.com.br; 4 Laboratório de Pesquisas do Cefores, Universidade Federal do Triângulo Mineiro, 38015-050 Uberaba, MG, Brazil; tppfarmacia@icloud.com; 5 Institute of Biological and Natural Sciences, Federal University of Triângulo Mineiro, 38015-050 Uberaba, MG, Brazil; siomars@gmail.com; 6 Clinical Hospital, Division of Endocrinology, Federal University of Triângulo Mineiro, 38025-180 Uberaba, MG, Brazil; elisabete@mednet.com.br; 7 Clinical Pathology Service, Microbiology, Federal University of Triângulo Mineiro, 38025-180 Uberaba, MG, Brazil; marcelo.costa2006@hotmail.com

**Keywords:** urinary infection, antibiotics, bacterial resistance, *Escherichia coli*

## Abstract

Empirical and prolonged antimicrobial treatment of urinary tract infections caused by *Escherichia coli* is associated with the emergence of bacterial resistance, and not all countries have strict policies against the indiscriminate use of drugs in order to prevent resistance. This cross-sectional and retrospective study (2010–2015) aimed to evaluate the sensitivity and resistance of patient-derived *E. coli* to different drugs broadly used to treat urinary infections in Brazil: ampicillin + sulbactam, cephalothin, ciprofloxacin, norfloxacin, and nitrofurantoin. We obtained 1654 *E. coli* samples from ambulatory patients with disease symptoms of the urinary tract from a Brazilian public hospital. While all antibiotics were effective in killing *E. coli* to a large degree, nitrofurantoin was the most effective, with fewer samples exhibiting antibiotic resistance. We assessed the costs of generic and brand name versions of each antibiotic. Nitrofurantoin, the most effective antibiotic, was the cheapest, followed by the fluoroquinolones (ciprofloxacin and norfloxacin), ampicillin + sulbactam and, lastly, cephalothin. Finally, assessment of antibiotic resistance to fluoroquinolones over the study period and extrapolation of the data led to the conclusion that these antibiotics could no longer be effective against *E. coli*-based urinary infections in approximately 20 years if their indiscriminate use in empirical treatment continues.

## 1. Introduction

Bacterial urinary infections are common in clinical practice, where approximately 80/1000 consultations lead to this diagnosis [1]. In general, infections are caused by Gram-negative aerobic bacteria of the intestinal microflora. The most predominant species is the enterobacterium *Escherichia coli*, which is normally associated with acute cases [1,2]. *E. coli* causes both intestinal and extra-intestinal infections. Antimicrobial treatment of infections caused by *E. coli* must be appropriately targeted; for this purpose, the medical community uses a wide variety of drugs both alone or in combination. Among the most widely used drugs or combinations in Brazil are ampicillin with sulbactam, cephalothin, ciprofloxacin, norfloxacin, and nitrofurantoin.

While antibiotics are considered the most effective method of treatment for bacterial infections, their empirical, indiscriminate, prolonged, or incorrect usage contributes significantly to the emergence of new infections by leading to the selection of resistant strains [3,4]. For instance, 20% of relapses and 3.7% of new cases of tuberculosis are caused by rifampicin- and isoniazid-resistant strains of *Mycobacterium tuberculosis* [5]. In addition, several other classes of bacteria have already exhibited multidrug resistance to antibiotics, such as *Klebsiella pneumoniae* and *E. coli* strains producing extended-spectrum beta-lactamase (ESBL), which hydrolyses the beta-lactam ring of penicillin, cephalosporins, and other related antibiotics, contributing to treatment failure [6,7]. Drug-resistant microorganisms have spread worldwide, affecting both hospital infections and the community in general. This highlights the diversity and potential genetic mechanisms of resistance, as well as indicating a co-selection to antibiotics, especially fluoroquinolones, aminoglycosides, and sulphonamides [8], drugs of choice for empirical treatment of urinary tract infections worldwide.

The prevalence of antibiotic resistance stems primarily from the promiscuous nature of bacteria, as susceptible bacteria may acquire resistance genes from plasmids and other horizontally transferred genetic material, resulting in evolution by leaps [9,10]. In addition to plasmid-mediated resistance mechanisms, there are other resistance factors associated with empirical treatment that are of concern, especially when considering urinary tract infections caused by *E. coli*. One such concern is the ability of bacteria to form micro-colonies (biofilms) on the mucosal lining of the bladder to resist the host’s immune response and conventional antibiotic treatment [11]. Two factors are associated with the development of these biofilms: the incorrect use of antibiotics to eliminate the infection [11] and the inappropriate choice of medicines due to individual and collective economic problems, especially in developing countries [12,13,14]. Several considerations are involved in the correct choice of antibiotics for the treatment of genitourinary tract infections, including pharmacokinetics, pharmacodynamics, costs, and the ability of the drug to eliminate the causative agent of the infection. Considerations, such as cost, may be one of the reasons that resistance levels vary greatly by country [3]. It is also worth mentioning that, as of yet, there are not many treatment alternatives for patients affected by resistant strains. Thus, understanding the problem of antibiotic resistance and determining the best antibiotic for treatment or discovering new treatment alternatives represents one of the greatest challenges of this century.

Our study used a cross-sectional, retrospective study to determine the antibiotic susceptibility profiles of 1654 samples positive for *E. coli* that were obtained from outpatients of a public hospital in the Brazilian Health System. In addition, we also surveyed and compared the real costs to the community of the primary antibiotics used to treat these infections.

## 2. Materials and Methods

### 2.1. Experimental Design

This retrospective, cross-sectional study was conducted in a public hospital at the Federal University of Triângulo Mineiro belonging to the Brazilian Health System in Minas Gerais/Brazil. This hospital attends to approximately 1.17 million inhabitants belonging to more than 27 cities in the macroregion south of the Triângulo Mineiro. Data were collected from January 2010 to December 2015 from the clinical pathology services database of the hospital, including reports of technical procedures conducted and urine cultures performed in the laboratory. The study was conducted in accordance with the Declaration of Helsinki, and the protocol was approved by the Ethics Committee of Federal University of Triângulo Mineiro (protocol number: 914797).

### 2.2. Inclusion and Exclusion Criteria

**Figure 1:**
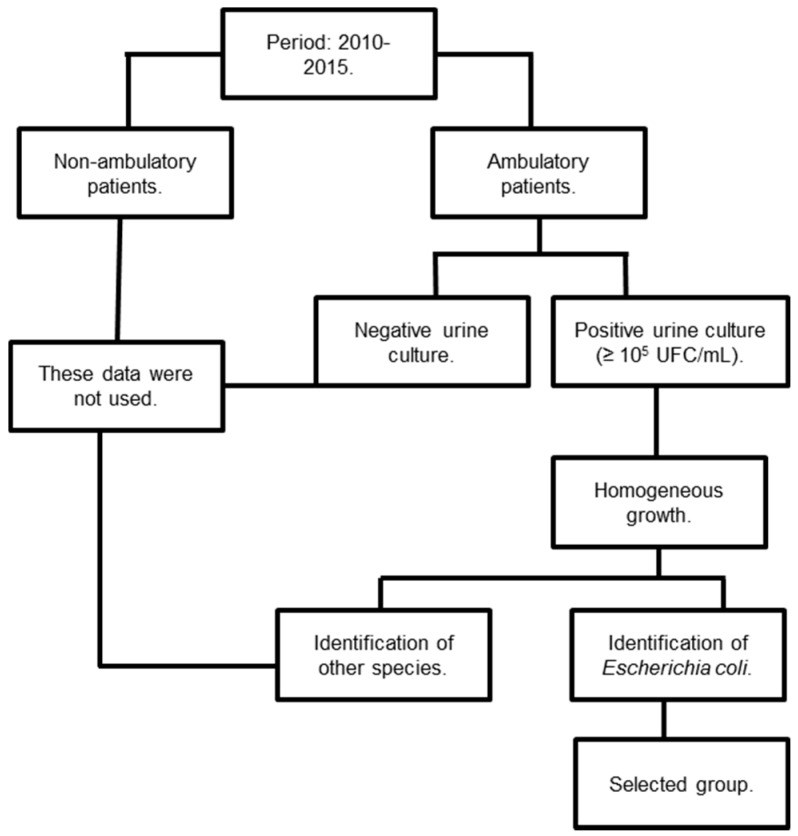
Representation of determination of adherence to criteria for inclusion or exclusion in the study.

Adherence to trial criteria was assessed in a systematic fashion, as represented in Figure 1. First, we only included ambulatory patients who underwent examination of urine culture during the trial period (January 2010 to December 2015). Furthermore, we only included positive urine cultures with ≥10^5^ colony-forming units (CFU)/mL, and we excluded urine cultures that were not positive for *Escherichia coli* after identification.

### 2.3. Bacterial Identification

Urine was sampled in a sterile container, and 0.001 mL was plated on cystine lactose electrolyte deficient (CLED) agar with a calibrated platinum loop. The culture was incubated for 18–24 h at 35 °C, and bacterial colonies were then counted, with results expressed in CFU/mL. Species identification was performed using standard biochemical tests. 

### 2.4. Antimicrobial Susceptibility Testing

Antimicrobial susceptibility testing was conducted on Mueller Hinton agar (HiMedia, Mumbai, India) using a standard disc diffusion method with discs of standard potency as per the Clinical and Laboratory Standards Institute’s guidelines [15]. The antibiotics tested were as follows (potency in μg/disc): ampicillin + sulbactam (10 + 10), cephalothin (30), ciprofloxacin (5), norfloxacin (also known as floxacin) (10), and nitrofurantoin (300). After evaluation of the antimicrobial susceptibility, we investigated the relative frequency (RF) of the number of susceptible or resistant cases in each year, where we obtained the ratio between the frequencies of susceptibility (S) and resistance (Res) (∑RFS)/(∑RFRes) in order to assess whether there was variation in the number of these cases/year depending on the antibiotic analyzed.

### 2.5. Commercial Values

To determine the costs of the investigated antibiotics for statistical comparison, we used standard prices from the *Journal of the Brazilian Association of Pharmaceutical Commerce*.

### 2.6. Statistical Analysis

Statistical analysis was performed using the GraphPad Prism program (GraphPad Software, San Diego, CA, USA). To analyze paired elements, we used the paired *t*-test. Normality (Kolmogorov-Smirnov) and homogeneity of variance (Bartlett’s) tests were applied to all variables. When under normal distribution and homogeneous variance, parametric tests (ANOVA with post-hoc Tukey’s multiple comparison) were applied, and the results are expressed as the mean ± SEM. Otherwise, we used non-parametric tests (Kruskal-Wallis test with Dunn’s multiple comparison), and the results are expressed as median, maximum, and minimum values. Differences were considered significant when *p* ≤ 0.05 [16].

## 3. Results

The frequency of outpatient urine samples found in the database that were positive for the bacteria *E. coli* was high (~79%) compared to the frequency of samples positive for other microorganisms. This frequency was also higher in female patients (83%) than in male patients (17%). Over the course of the six years of study, we obtained 1654 *E. coli*-positive urine samples, with an average of 275.70 ± 30.16 samples/year and a coefficient of variation of 26.80% (Table 1). 

### 3.1. Efficacy of Antibiotics Against Patient Uroculture-Derived E. coli Samples

**Figure 2:**
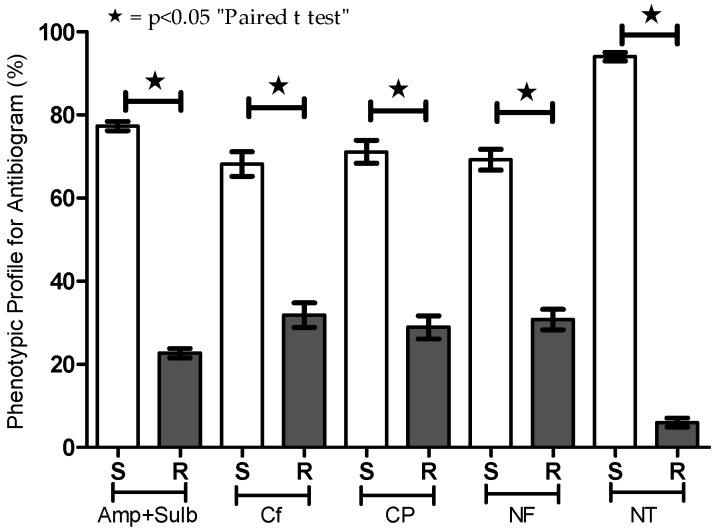
Phenotypic profile for susceptibility testing (antibiogram) of urine cultures positive for *E. coli* from outpatients. Antibiotics including ampicillin + sulbactam (Amp + Sulb), cephalothin (CF), ciprofloxacin (CP), norfloxacin (NF), and nitrofurantoin (NT) were tested by the disc diffusion method. Percentages of sensitive (S) and resistant (R) strains are presented. * *p* < 0.05.

For each *E. coli*-positive urine sample, we evaluated the phenotypic profile of sensitivity and resistance to five antibiotics (Table 1 and Figure 2). Although there was variation in the distribution between the sensitivity and resistance samples (ampicillin + sulbactam = 77.32/22.68; cephalothin = 68.17/31.83; ciprofloxacin = 71.10/28.90; norfloxacin = 69.25/30.75; nitrofurantoin = 94.04/5.95), all antibiotics tested exhibited significant efficacy (*p* < 0.05) (Figure 2).

### 3.2. Relative Frequency of Antibiotics Used for Empirical Treatment of Urinary Infections

After verifying that all tested antibiotics were effective against patient-derived *E. coli*, we evaluated whether there were significant differences in the distribution in the ratio of the relative frequencies of susceptibility and resistance over the years to each antibiotic. In general, ratios higher than 1 (>1) in the relative frequencies demonstrate positive indices for antimicrobial susceptibility (Table 2). In this sense, the antibiotic nitrofurantoin was the one who showed the best results once it exceeded five-fold of the ratio of the relative frequency of susceptibility and resistance over the years (*p* < 0.05). On the other hand, the antibiotics of the classes of fluoquinolones and cephalothin have ratios of the relative frequencies lower than 1, indicating less satisfactory indices for antimicrobial susceptibility (Table 2). In other words, regardless of the year in which the test was performed, the frequency of susceptible samples (relative frequency) was higher for the nitrofurantoin. Likewise, the frequency of resistant samples per year was also always lower for the nitrofurantoin.

### 3.3. Temporal Variation in Antimicrobial Resistance in Brazil

**Figure 3:**
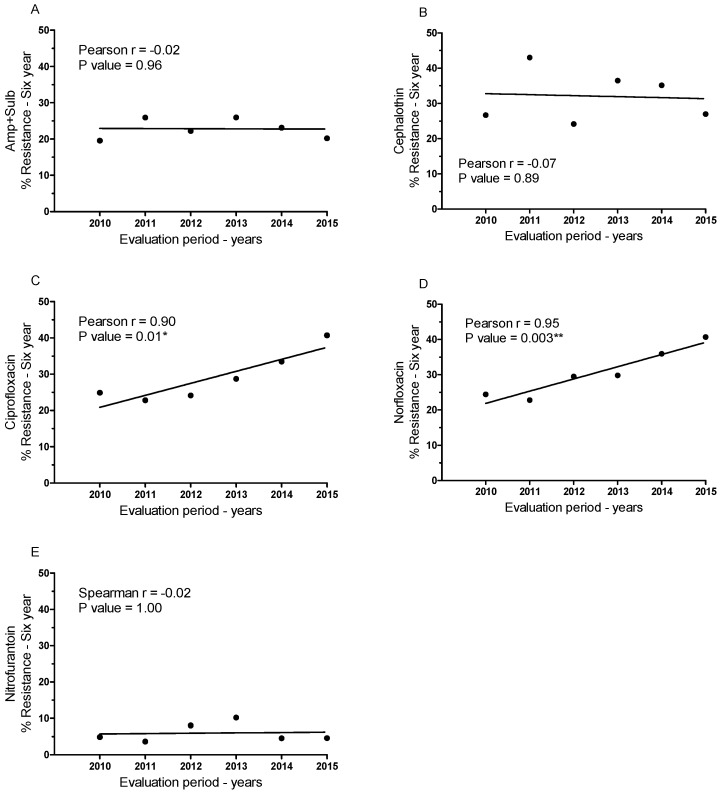
Percentage of resistant patient-derived *E. coli* samples over the six-year study period for each antibiotic assessed and Pearson’s correlation of resistant samples over time: (**A**) ampicillin + sulbactam, (**B**) cephalothin, (**C**) ciprofloxacin, (**D**) norfloxacin, and (**E**) nitrofurantoin. * *p* < 0.05; ** *p* < 0.005.

In addition to assessing the susceptibility of samples to different antibiotics, we also investigated temporal variation in antibiotic resistance (Figure 3 and Table 3). The percentage of samples resistant to each antibiotic in each of the six years was plotted, and Pearson’s correlation was determined (Figure 3A–E). The data reveal non-linear and non-significant correlations for ampicillin + sulbactam, cephalothin, and nitrofurantoin (Figure 3A,B,E). However, there were consistent linear increases in resistance to ciprofloxacin and norfloxacin, both fluoroquinolones (Figure 3C,D). These increases were in the order of 63.53% for ciprofloxacin and 66.50% for norfloxacin from 2010 to 2015. Both ciprofloxacin and norfloxacin exhibited significant positive correlations between the resistance of samples and the year the samples were collected (*p* < 0.05).

We extrapolated this correlation into an estimation of future resistance to fluoroquinolones (Table 3). Based on this prediction, we can assume that if resistance continues to increase at the same pace, fluoroquinolones will no longer be effective against urinary tract infections 20 years from now.

### 3.4. Variation in the Cost of Drugs Used to Treat Genitourinary Infections Caused by E. coli

An important factor in successful treatment of bacterial infections is the implementation of the treatment, beginning with the acquisition of the drug and followed by its correct usage. Thus, we conducted a survey of the cost of drugs used to treat urinary infections caused by *E. coli*. Most of these drugs are available from community clinicians, with the exception of cephalothin, which is restricted to hospital use. Values take into account whether the medicine is generic or brand-name. The only exception was Sulbamox BD^®^ (Farmasa, Sao Paulo, Brazil), which was compared to another generic drug of the same class (β-lactamase inhibitor) because it is generally prescribed. The data revealed high variation in cost (amounts in Brazilian currency, the real) based on antibiotic, brand, and tax rate of the state (12%, 17%, 18%, or 19%). Costs ranged from R$6.17 ± 0.11 to R$230.80 ± 4.17 for drugs used in the community and up to R$244 ± 4.41 for drugs used only in the hospital (Table 4).

We calculated the average commercial value of brand-name and generic drugs for each antibiotic used for treatment of genitourinary tract infections caused by *E. coli* (Table 4), revealing significant differences between the means (*p* < 0.05). Surprisingly, while nitrofurantoin was demonstrated to be the most effective by our susceptibility testing, it exhibited the lowest average cost (R$7.83 ± 1.66) among all evaluated antibiotics (*p* < 0.05).

## 4. Discussion

In this study, we evaluated the efficacy of antibiotics against strains of *E. coli* causing urinary tract infections. We also performed a comparison of the actual costs of the generic and brand-name antibiotics used for the treatment of urinary infections.

After assessing the bacterial strains associated with urinary tract infection in ambulatory patients over six years, we noted a high frequency of infections caused by *E. coli*, particularly in female patients. This is corroborated by data on *E. coli* infections worldwide [17,18]. We believe that *E. coli* infection may be even more prevalent considering the large number of cases of urinary infection where the causative agent is never identified and/or the infection is treated empirically [19]. Moreover, although the number of *E. coli*-positive samples identified in our study was considerable (1654 positive samples over six years, with an average of 276 new cases annually), *E. coli* infection is likely to be even more prevalent, as these numbers do not include outpatients, in whom urinary tract infections may be common.

Several studies have evaluated the microbicidal activity of the antibiotics used for urinary tract infections, as well as the mechanisms involved in drug resistance [6,18,20,21]. Such studies demonstrate a major cause for concern regarding the epidemiology of resistance to antibiotics used to treat urinary infections [22,23]. In our first experiment, we measured the effectiveness of five antibiotics used to treat urinary infections: ampicillin combined with sulbactam, an inhibitor of beta-lactamase; cephalothin; the fluoroquinolones ciprofloxacin and norfloxacin; and nitrofurantoin. In general, all antibiotics exhibited high efficacy, indicating that they are appropriate for empirical treatment according to each treatment specification [24,25]. However, we also observed some variation in the antibiotic efficacy of different antibiotics.

Thus, we evaluated the susceptibility and resistance profiles separately for each antibiotic. Nitrofurantoin was the most effective treatment against *E. coli* isolated from the urine samples and presented the lowest resistance rate. Nitrofurantoin was followed by ampicillin + sulbactam, the fluoroquinolones and, lastly, cephalothin, in terms of efficacy. In assessing antibiotic efficacy over the period of the six years of the study, we observed a significant increase in resistance to fluoroquinolones (ciprofloxacin and norfloxacin) over time, which may be due to empirical treatment of urinary infections. This effect was also reported in Uruguay, a country with extensive use of fluoroquinolones as a form of empirical treatment for urinary infections [21], as well as in another study highlighting the risk factors for urinary tract infections in ambulatory patients exhibiting microbial resistance to fluoroquinolones [26]. The concern regarding microbiological resistance requires changes enabling preventive measures or the discovery of new therapeutic targets by the scientific community. This is mainly owed to the studies showing a close relationship between the development of mechanisms of resistance and strategies that have been adopted for decades to restrain infectious processes [27,28]. Evidence of this problem are related to the increasing frequency of Enterobacteriaceae resistant to to β-lactamics or carbapenemics observed in recent years, which has also been extended to aminoglycosides and fluoroquinolones [29]. Our data corroborate the findings of these studies, as fluoroquinolones are the treatment of choice for urinary infections in Brazilian states and are thus susceptible to factors associated with microbial resistance. Most importantly, we used the data on resistance to fluoroquinolones over the last six years to extrapolate the pattern of microbial resistance in the future. By this estimation, we have deduced that fluoroquinolones will no longer be effective against *E. coli-*based urinary infections in approximately 20 years if appropriate measures are not taken against the generalized misuse of antibiotics in empirical treatments.

One of the major reasons for such increases in resistance is the misapplication of antibiotics due to economic and social issues in some countries [12,13,14]. We, therefore, investigated the costs of brand-name and generic versions of the five different antibiotics and observed discrepancies between the costs of various antibiotics. The costs of the drugs are not only based on their efficacy, but are also related to the cost of production by different pharmaceutical companies. This discrepancy, assessed by obtaining the mean of generic and brand-name drugs, extended to all antibiotics tested. Our results revealed a significantly lower cost for the acquisition of nitrofurantoin, followed by norfloxacin, ampicillin + sulbactam, ciprofloxacin and, finally, cephalothin. Costs for the generic versions of ampicillin + sulbactam and ciprofloxacin were not significantly different. Thus, our findings highlight the importance taking into account both the effectiveness of an antibiotic treatment and the cost for patients. Simoens [13] has discussed this topic, stating that physicians must consider these and others factors when prescribing an antibiotic and determining whether a specific antibiotic treatment adds enough value to justify extra costs. Still, in the 1980s, two aspects concerning microbial resistance were considered more relevant: the indication of inappropriate antibiotics for patients with nosocomial infections and the cost of antibiotics, where patients could receive antibiotics at lower cost [27]. In this same study, the authors also pointed out that a high rate of subjects receiving antibiotic therapy with lower cost and, therefore, the implementation of computer programs with applications in hospital surveillance, would be necessary.

Indeed, in addition to the misapplication of antibiotics due to economic and social issues, the elevated number of mutations in bacteria is a natural and inseparable factor for the occurrence of resistance. Hence, it is crucial to discuss the best approaches to choose which antibiotic will be used in the future, especially because there is an inverse relationship between the necessity of new antibiotics and the amount of newly discovered drugs of this class. The results presented here are of major concern and a matter of debate for everyone involved in healthcare, especially considering the use of fluoroquinolones for the treatment of *E. coli*-based urinary infections. Clinicians must be careful regarding their choice of antibiotics considering costs and the occurrence of microbial resistance. Taken together these strategies may contribute to a more effective control of infections, thus reducing loss of life. Finally, new strategies aiming the treatment and control of bacterial infections are still needed. These approaches may include the discovery of new medicines, vaccines, the control of hospital infections, and the rational use of existing antibiotics.

## 5. Conclusions

In summary, our data reveal the importance of evaluating the antibiotic susceptibility profile of patient *E. coli* samples in order to obtain the best strategy for empirical treatment of urinary infections. We recommend that clinicians take into account not only the effectiveness of specific antibiotics but also the actual costs to the patient when determining the best course of treatment.

## Figures and Tables

**Table 1:** Absolute values of antimicrobial sensitivity in urocultures positive for *E. coli* from outpatients, 2010–2015.

Year	Amp + Sulb		Cephalothin		Ciprofloxacin		Norfloxacin		Nitrofurantoin		N
	S	R	S	R	S	R	S	R	S	R	
2010	181	44	165	60	169	56	170	55	214	11	225
2011	143	50	110	83	149	44	149	44	186	7	193
2012	203	58	198	63	198	63	184	77	240	21	261
2013	268	94	230	132	258	104	254	108	325	37	362
2014	196	56	167	85	171	81	165	87	241	11	242
2015	296	75	271	100	220	151	220	151	354	17	371
Total											1654

Amp + Sulb = ampicillin + sulbactam; N = samples/year; S = susceptibility; R = resistance.

**Table 2:** Ratio of the relative frequencies of susceptibility and resistance in urocultures positive for *E. coli* from outpatients, 2010–2015.

Years	(∑RFS)/(∑RFRes)	Ratio of Relative Frequency (S/Res)/Antibiotic				
		Amp + Sulb	Cephalothin	Ciprofloxacin	Norfloxacin	Nitrofurantoin
2010	1	1.03	0.69	0.77	0.77	4.94
2011	1	0.90	0.42	1.03	1.03	7.47
2012	1	0.89	0.82	0.82	0.82	2.79
2013	1	1.01	0.61	0.88	0.88	2.96
2014	1	1.17	0.67	0.70	0.70	6.41
2015	1	1.43	0.98	0.53	0.53	7.56
Ẋ ± Std. Error	-	1.07 ± 0.08 ^a^	0.70 ± 0.07 ^b^	0.79 ± 0.07 ^a,b^	0.79 ± 0.07 ^a,b^	5.35 ± 0.87 ^c^

S = sensibility; Res = resistance; RF = relative frequency; Amp + Sulb = ampicillin + sulbactam; Ẋ = mean; Std. Error = standard error. Lowercase letters indicate significant differences according to Tukey’s multiple comparison test (*p* < 0.05). In ^a^, Amp + Sulb is different of Cephalothin and Nitrofurantoin. In ^b^, Cephalothin is different of Amp+Sulb and Nitrofurantoin. In ^a,b^ Fluoroquinolones are only different of nitrofurantoin. In ^c^, Nitrofurantoin is different from all other tested antibiotics.

**Table 3:** Estimation of the total resistance to Fluoroquinolones in Brazil based on the empirical treatment of patients with urinary infections.

Fluoroquinolones	Resistance to Fluoroquinolones (%)					
	50	60	70	80	90	100
Norfloxacin (Year)	2017	2020	2023	2026	2029	2032
Ciprofloxacin (Year)	2018	2021	2024	2027	2030	2033

**Table 4:** Cost of antibiotics at different tax rates.

Product (Manufacturer)	Presentation	MPC (R$)				Mean ± SE (MPC)	Mean ± SE (GE-Brand)
		12%	17%	18%	19%		
Sulbamox BD^®^ (Farmasa)	875 + 125 mg—14 cpr	72.00	76.34	77.27	78.22	75.96 ± 1.37	84.60 ± 8.64
Amoxicilina + clav K (Eurof.)	875 + 125 mg—14 cpr	88.38	93.71	94.85	96.01	93.24 ± 1.69	
Cephalothin (Ariston)	1g inj ct 50 un	231.33	245.28	248.26	251.31	244.00 ± 4.41	244.00
Cephalothin (Novafarma)	1g inj ct 50 un	231.3	245.23	248.23	251.28	244.00 ± 4.41	
Cipro^®^ (Bayer)	500 mg—14 cpr	218.74	231.93	234.75	237.64	230.80 ± 4.17	136.50 ± 94.28
Clor. Ciprofloxacin (Medley)	500 mg—14 cpr	40.04	42.46	42.97	43.5	42.24 ± 0.76	
Floxacin^®^ (Merck Sharp)	400 mg—14 cpr	30.25	32.07	32.46	32.86	31.91 ± 0.58	31.92 ± 0.01
Norfloxacin (Medley)	400 mg—14 cpr	30.26	32.09	32.48	32.88	31.93 ± 0.58	
Macrodantina^®^ (Sc.P)	100 mg—28 cpr	8.99	9.54	9.65	9.77	9.49 ± 0.17	7.83 ± 1.66
Nitrofurantoin (Teuto)	100 mg—28 cpr	5.85	6.2	6.27	6.35	6.17 ± 0.11	

MPC = maximum price to consumers; GE = generic; cpr = tablets; un = unity; Eurof. = Eurofarma; Sc.P = Schering Plough.
